# Diesel exhaust increases EGFR and phosphorylated C-terminal Tyr 1173 in the bronchial epithelium

**DOI:** 10.1186/1743-8977-5-8

**Published:** 2008-05-06

**Authors:** Jamshid Pourazar, Anders Blomberg, Frank J Kelly, Donna E Davies, Susan J Wilson, Stephen T Holgate, Thomas Sandström

**Affiliations:** 1 Department of Respiratory Medicine and Allergy, University Hospital, Umeå, Sweden; 2 Lung Biology, Pharmaceutical Sciences Research Division, School of Biomedical and Health Sciences, King's College London, London, UK; 3 Allergy and Inflammation Research, University of Southampton, Southampton, UK

## Abstract

**Background:**

Epidemiological studies have demonstrated adverse health effects of environmental pollution. Diesel exhaust (DE) is a major contributor to particulate matter pollution. DE exposure has been shown to induce a pronounced inflammatory response in the airways, together with an enhanced epithelial expression of cytokines such as IL-8, Gro-α, IL-13 and activation of redox sensitive transcription factors (NFκB, AP-1), and MAP kinases (p38, JNK). The aim of the present investigation was to elucidate the involvement of the epidermal growth factor receptor (EGFR) signalling pathway in the epithelial response to DE *in-vivo*.

**Results:**

Immunohistochemical staining was used to quantify the expression of the EGFR, phosphorylated Tyrosine residues, MEK and ERK in the bronchial epithelium of archived biopsies from 15 healthy subjects following exposure to DE (PM_10_, 300 μg/m^3^) and air. DE induced a significant increases in the expression of EGFR (p = 0.004) and phosphorylated C-terminal Tyr 1173 (p = 0.02). Other investigated EGFR tyrosine residues, Src related tyrosine (Tyr 416), MEK and ERK pathway were not changed significantly by DE.

**Conclusion:**

Exposure to DE (PM_10_, 300 μg/m^3^) caused enhanced EGFR expression and phosphorylation of the tyrosine residue (Tyr 1173) which is in accordance with the previously demonstrated activation of the JNK, AP-1, p38 MAPK and NFkB pathways and associated downstream signalling and cytokine production. No effects were seen on the MEK and ERK pathway suggesting that at the investigated time point (6 hours post exposure) there was no proliferative/differentiation signalling in the bronchial epithelium. The present findings suggest a key role for EGFR in the bronchial response to diesel exhaust.

## Introduction

Numerous studies have reported an association between increased ambient levels of particulate matter (PM) pollution and increased respiratory and cardiovascular morbidity as well as mortality [1,2]. Diesel engine exhaust (DE) is a major contributor to ambient PM pollution and diesel engines may produce ten times or more nanometer-sized particles (nanoparticles) compared to gasoline engines. Diesel exhaust particles (DEP) have been shown to have substantial toxicological capacity, associated with particle size and surface chemistry characteristics, including metal and organic components with oxidative capability [3-6].

Mechanistic aspects of DE exposure in humans have been addressed in a series of experimental studies [7-12]. Changes in the production of IL-8, IL-10, IL-13 and Gro-α in the bronchial epithelium as well as an upregulation in the expression of the vascular endothelial adhesion molecules ICAM-1 and VCAM-1 have been demonstrated. These findings were accompanied by a pronounced inflammatory cell infiltration, including activated neutrophils, lymphocytes and mast cells in the bronchial mucosa [7,9-12] as well as generation of reactive oxygen species (ROS) and signs of oxidative stress [8]. Of note, asthmatic subjects have an enhanced sensitivity to PM air pollution [1,13] while having an compromised oxidative defence capacity. Asthmatics also have a different inflammatory response to DE than healthy subjects and develop increased bronchial hyperresponsiveness following challenge [12,14].

Bronchial mucosal biopsies, sampled after air and DE exposures in healthy humans, have been instrumental in determining the epithelial expression of redox sensitive mitogen-activated protein kinases (MAPKs) and transcription factors involved in the regulation of airway inflammation. Using this approach it was demonstrated that DE activates the p38 and JNK MAPK pathways and leads to increased expression of the NFκB and AP-1 transcription factors, associated with findings of downstream cytokine production [9,11,15]. Receptor tyrosine kinases (RTKs), including epidermal growth factor receptor (EGFR), are primary mediators of external stimuli and incoming signals. EGFR has been demonstrated to play a key role in bronchial epithelial repair, remodelling and control of airway inflammation. It achieves this by regulating a range of cellular processes including mitogenesis, apoptosis, migration, differentiation and proliferation, all of which are of important in many situations and conditions, including asthma. Furthermore, EGFR activation by metals and hydrocarbons with oxidative capacity has been shown to activate downstream MAPkinases and transcription factors [16-18].

In the present study, we therefore sought to investigate the hypothesis that the activation of transcription factors and MAP kinases and increased downstream production of cytokines observed in bronchial mucosal biopsies following DE challenge in human subjects was accompanied by activation of upstream pathways such as EGFR and phosphorylation or transphosphorylation of specific tyrosine residues of EGFR such as Tyr 845, Tyr 992, Tyr 1068, Tyr 1110 and Tyr 1173. In addition, we investigated whether EGFR activation by diesel exposure could be mediated by Src activation and phosphorylation of Src Tyr 416 and leading to transactivation of EGFR at Tyr 845 and whether activation of EGFR would increase the downstream MEK-ERK pathway signalling, linked to proliferation and differentiation.

## Results

**Figure 1:**
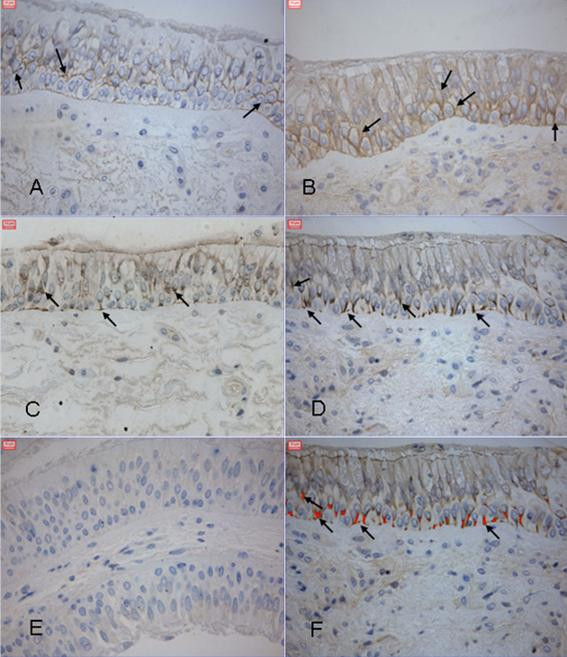
**Immunostaining**. Immunoreactivity within the bronchial epithelium for EGFR after air and diesel exhaust exposure. Positive staining (brown) can be seen on the basolateral border of the columnar cells after exposure to air (**panel A**) and throughout the epithelium following exposure to diesel exhaust (**panel B**). Arrows show intracytoplasmatic Tyr 1173 immunostaining in the basoperinuclear region of columnar cells and basolateral border of basal cells (**panel C **After air exposure and **panel D **after DE exposure). **Panel E: **Primary IgG antibody serving as a negative contol. **Panel F **demonstrates the normal brown DAB staining in **panel D **which is presented by red color following computer-aided selection and quantification by the image analyser program.

The immunoreactivity for EGFR was evident on the basolateral border of the columnar cell (figure 1A) when subjects were exposed to air. Following exposure to DE, expression could be observed throughout the epithelial layer (figure 1B). Immunostaining of phosphorylated Tyr 1173 was intracytoplasmatic in the baso-perinuclear region of the columnar cells and on the basolateral borders of the basal cells in the bronchial epithelium after exposure to both air and DE (figure 1C and 1D respectively).

Short term exposure to diesel exhaust induced a significant increase in the expression of EGFR in the bronchial epithelium; 0.69% (median) of the total epithelial area compared to 0.24% after air exposure (p = 0.004). This change was accompanied by an increased phosphorylation of Tyr 1173; 3.2% after diesel exhaust exposure vs. 2.2% after air (p = 0.02). The expression of other EGFR tyrosine residues such as Tyr 845, Tyr 992, Tyr 1068 and Tyr 1110 and Src related tyrosine (Tyr416) and the ERK pathway (MEK and ERK) were not significantly altered after diesel exposure (Table 1)

**Table 1:** Immunoractivity of investigated antibodies in 15 Air and DE paired subjects participated in study.

**Immunoreactivity**	**Air**	**Diesel**	**p value**
EGFR	0.240 (0.100–0.370)	0.690 (0.210–1.960)	**p = 0.004**
Tyr 845	0.220 (0.027–0.520)	0.460 (0.160–1.030)	p = 0.1
Tyr 992	0.450 (0.320–0.580)	0.530 (0.220–1.540)	p = 0.14
Tyr 1068	0.015 (0.002–0.250)	0.180 (0.013–0.550)	p = 0.18
Tyr 1110	1.090 (0.560–2.140)	1.780 (0.980–2.200)	p = 0.39
Tyr 1173	2.230 (1.380–2.890)	3.160 (1.800–5.070)	**p = 0.02**
Tyr 416	0.033 (0.001–0.120)	0.016 (0.003–0.800)	p = 0.13
MEK 1, 2	1.330 (0.310–2.670)	1.410 (0.630–4.440)	p = 0.28
ERK 1, 2	0.140 (0.024–0.320)	0.160 (0.045–0.650)	p = 0.14

Two sections from each biopsy and exposure were evaluated during investigation of each antibody.

Bronchial epithelium expression of EGFR, Tyrosine sites and downstream signalling immunoreactivity is given as percentage (%) positively stained area of the total measured epithelial area. All values are quoted as medians with inter-quartile range. Statistical comparisons by Wilcoxon's paired rank test.

## Discussion

Diesel engine exhaust has been demonstrated to induce inflammation in the bronchial epithelium, which apart from its classical barrier function, increasingly has been demonstrated to carry important immune regulatory properties. The EGFR has been shown to be of importance in these principal functions, as highlighted in respiratory diseases such as asthma, the most common condition recognised to be affected by particulate air pollution [19-22]. In this first *in vivo *study examining the involvement of EGFR in the human airways responses to DE, analyses of bronchial mucosal biopsies demonstrated a significantly increased expression of EGFR in the bronchial epithelium six hours after challenge. This was associated with a significantly increased phosphorylation of the Tyr 1173 auto-phosphorylation site on the EGFR C-terminal. Src was not found to be involved in the EGFR activation as indicated by unchanged phosphorylation of Src Tyr 416 and EGFR Tyr 845. At this time post DE exposure, the EGFR downstream MEK/ERK signalling pathway was also unaffected.

It is recognized that certain transcription factors, which regulate cytokine production and inflammatory responses may be activated by MAPKs and their upstream signalling pathways. This has been confirmed to take place in the human airway epithelium after DE exposure, by analyses of archived human bronchial biopsies [15]. The nuclear expression of phosphorylated p38 MAPK was significantly upregulated, together with increased nuclear translocation of p-JNK MAPK and c-jun of the AP-1 complex. Additionally, the nuclear expression of NFκB was increased. These transcription factors are known to control the production of a wide range of cytokines. DE exposure has been shown to enhance the production of IL-8 and Gro-α in the bronchial epithelium, accompanied by a pronounced neutrophil influx in the bronchial wall. This neutrophilia was mediated by the upregulation of the vascular adhesion molecule expression of P-selectin and ICAM-1 [10-12]. Furthermore, a DE mediated increase in the bronchial epithelial expression of IL-13 has been reported [9]. When considering upstream signalling and regulation of these signal transduction pathways, it is clear that EGFR could have the potential to regulate, or assist in regulating, all the above mentioned events. Therefore a critical question to address was whether activation of EGFR would occur after inhalation of diesel exhaust, which is a complex mixture of gases and particulates.

Most likely, nitrogen dioxide (NO_2_), a major gaseous component in DE does not play any major role in the bronchial mucosal effects of DE in humans [23]. In contrast to DE, exposure to NO_2 _at levels similar or higher than employed here, has failed to elicit any bronchial mucosal inflammatory responses [23]. Indeed, there are several indications in the literature that metal and hydrocarbon induced oxidative stress in the airways is involved in the airway response to DE. Even though the metal content in DEP is usually not as extensive as in residual oil fly ash (ROFA), it is clear that the content of transition metals may contribute to oxidative stress [8].

Oxidative stress related to hydrocarbons has *de facto *been suggested to be a common denominator for many DEP-induced cellular effects. Reactive oxygen species (ROS) can be produced following metabolism and bioactivation of hydrocarbons by cytochrome P-450 1A1 (CYP1A1). The polar quinones have the capacity to generate oxidative stress by redox cycling. Additionally, the metabolism of semiquinones by NADPH-cytrochrome P-450 reductase followed by autooxidation also results in ROS production [24-26]. Development of oxidative stress in the airways following DE challenge has been implied by analyses of human airway lavage samples [8]. Furthermore, genetic polymorphisms in Glutathione-S-transferases (GSTs) have been indicated to be critical for the defence against ROS and detoxification of DEP. The GSTM1 and GSTP1 genotypes have been confirmed to modify the allergen response by DEP in the nose in human subjects [27]. There are several studies indicating that EGFR is activated by metals, organic components and oxidative stress, supporting the notion that this receptor tyrosine kinase may play a major regulatory role in the inflammatory response to DE exposure [16,18,28]. While only providing indirect evidence, two recent studies have more specifically investigated the role of EGFR after exposure to DEP *in-vitro *[29,30]. In these studies, the authors were able to demonstrate that DEP triggered the secretion of amphiregulin, a ligand of EGFR, from bronchial epithelial cells, which could be blocked by ERK and EGFR tyrosine kinase inhibition as well as antioxidant supplementation. Furthermore, DEP quinone compounds have been shown to induce contraction of smooth muscle cells, mediated by activated phospholipase A2. This signalling pathway could be blocked by PTK and EGFR inhibitors. Taken together, these studies demonstrate the ability of DEP to both activate and transactivate EGFR.

**Figure 2:**
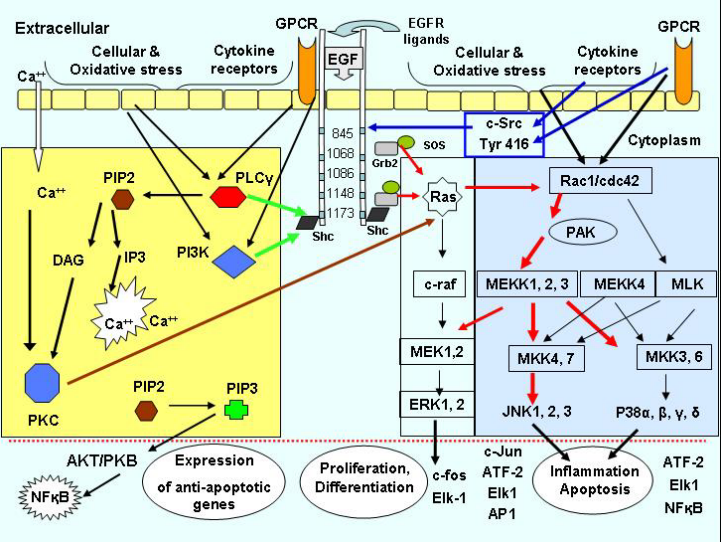
**Description of possible involved signalling pathways**. Schematically description of possible involved signalling pathways in the activation and phosphorylation of EGFR, protein tyrosine kinases and downstream signalling following DE exposure. Extended and modified from Puddicombe SM et al. Clinical and Experimental Allergy, 2000, 30, 7–11. The graph is inserted to allow for better understanding of the pathways and mediators discussed in this paper and is by no means conclusive. The yellow box shows the PTK activation pathway through cellular and oxidative stress, cytokine receptors and GPCR. N-SH2 domian in PLC-gamma recognize Tyr 1173 and is primary contributor to PLC-gamma association with the EGFR. Moreover stimulation of various GPCRs such as lysophosphatic acid (LPA) and endothelin-1 (ET-1) rapidly induce phosphorylation of adaptor protein Shc and Shc – Grb2 complex formation. Tyr 1173 on EGFR can function as a docking site for PLC-gamma and PI3-K (green arrow from yellow box). Furthermore Tyr 1173 interact with both SH2 and PTB domains in adoptor protein Shc plays major role in the Ras signalling and subsequence the EGF mediated activation of JNK which is dependent on Shc and controlling JNK activation and possible signalling pathways involved in MEKK1 activation and regulation of JNK and p38 MAPKs after EGF stimulation (red arrows show the signalling direction in the blue box).

EGFR has an extracellular ligand-binding domain, a membrane-spanning domain and a cytoplasmatic protein tyrosine kinase domain with a carboxyl terminal that contains tyrosine residues that undergo autophosphorylation during receptor activation [31-34]. Three major tyrosine sites, Y1068, Y1173 and Y1148 and two minor tyrosine sites, Y992 and Y1086, serve as sites of autophosphorylation following ligand binding or transphosphorylation by other stimuli (figure 2). These autophosporylation sites function as binding sites for Src homology 2 (SH2) and protein tyrosine binding (PTB) domains of a variety of signalling proteins with enzyme activity such as phospholipase C-γ (PLC-γ), signal transducers such as PI3-K and adaptor proteins such as Growth factor receptor-binding protein 2 (Grb2) and Src-homology and collagen protein (Shc). These create binding sites for SH2 or protein tyrosine binding (PTB) domains of proteins or adaptor molecules that link EGFR activation to the downstream signalling pathway.

Following ligand binding, epidermal growth factor (EGF), transforming growth factor-α (TGF-α) and amphiregulin may cause downstream activation of the Ras/MAPK pathway. Importantly, EGFR transduces not only its own ligands, but also various stimuli, such as cytokines via cytokine receptors and/or G-protein-coupled receptor activation, as well as oxidative stress all of which result in transactivation. The increased EGFR expression demonstrated after DE in this study is expected to be related to a ligand – activated receptor and inhibition of endocytosis and degradation of receptor which leads to increased receptor expression.

The observation of enhanced phosphorylation of Tyr 1173 is in accordance with the previously demonstrated DE-induced increase in epithelial expression of NFkB, JNK, c-jun and p38 MAPK together with cytokines under their regulation such as IL-8 and GRO-α. Increased phosphorylated Tyr 1173 can function as a docking site for signalling systems such as PLC-γ or PI3-K (figure 2, yellow box) and exert oxidative responses following DE exposure, linking to NFkB as well as Ras activation [35,36]. Tyr 1173 is suggested to be the primary autophosphorylated site that is involved in the PLC-γ association with EGFR [37], and that signal transduction across membrane coupled to PI3-Kinase may be involved in the activation of PLC-γ [38]. The autophosphorylated Tyr1173 also acts as a docking site for Shc (Src-homology and collagen protein) which in turn can bind to Grb2 (Growth factor receptor-binding protein 2) and build Shc-Grb2-Sos complex which leads to downstream activation [39,40].

The adaptor protein Shc binding to Grb2, complex building and subsequence MEK kinase-1 (MEKK1) activation may specifically regulate the demonstrated JNK and p38 MAPK activation (figure 2 blue box). It has been suggested that EGFR mediated JNK signalling is regulated by Shc and a transient interaction of Grb2 and MEKK1 [39,41]. In contrast, ERK pathway induction is depending on binding of the Grb2-Sos complex to Tyr 1068 and mediated by MEK1 phosphorylation, which was not increased in the present material [42,43]. The ERK pathway was expected to be upregulated in the present diesel challenge scenario, as were the JNK and p38 MAPK pathways. ERK activation transduces proliferative and differentiation responses which could be in demand after diesel challenge. The absence of ERK pathway activation contrasts with that seen in a recent study by Blanchet *et al*, [29] who demonstrated PM2.5 and archived DEP to cause specific ERK activation, with amphiregulin secretion, by use of different blocking agents for the MAPK pathways. Amphiregulin is an EGFR ligand known to contribute to GM-CSF release, which can be important for sustaining a proinflammatory response. Analyses of the present biopsy material not only failed to show any DE-induced increase in ERK activation but, as previously reported, GM-CSF expression was unaltered [11]. The difference between this study and that of Blanchet et al, may be due to a dose threshold effect. Another possibility is the time course of events, since in the current study bronchial mucosal biopsies were sampled at 6 hours after in-vivo DE exposure and Blanchet *et al *determined the in-vitro response in the 16-HBE cells after 18 hours and in absence of cooperation with other signalling involved in an *in-vivo *system. The question whether the ERK pathway is activated at a later time point after in-vivo diesel exposure in humans *in-vivo*, will shortly be addressed in archived biopsies sampled 18 hours post exposure [7]. Of even more importance, EGFR pathway activation after DE exposure, can also be addressed in archived biopsies from asthmatic subjects [12], in which EGFR pathways are of major importance in terms of epithelial barrier integrity, airway remodelling and signal transduction [21,44].

To examine the potential role of metals in DE induced EGFR activation, cellular PTK or non-receptor protein tyrosine kinase such as Src were considered. Src can act as co-transducer of EGFR signals and has been demonstrated to be involved in the responses of Zn^2+^-induced Ras activation via the EGFR. Src-dependent EGFR signalling has been reported to be mediated by phosphorylation of Tyr 845 and Tyr 1101 [45,46]. In this study we did not find any changes either in Tyr 845 (EGFR tyrosine) or Tyr 416, Src related tyrosine (which is an autophosphorylation site on c-Src). Even though a role for Src in transphosphorylating EGFR tyrosine in the time course after a DE exposure *in-vivo *cannot completely be ruled out, the present study has not given any support for its involvement at the 6 hour post exposure sampling time.

IL-13 is a Th_2 _cytokine that has been implicated in allergy and asthma airway inflammation, airway remodelling and bronchial hyperresponsiveness. Increased bronchial epithelial expression of IL-13 has previously been shown after DE exposure in healthy non-atopic subjects [9]. This response could potentially have been mediated by EGFR downstream, such as via AP-1. Interestingly, IL-13 and EGFR may interact in epithelial and goblet cell regulation. IL-13 has been shown to activate neutrophils, and may by interaction with EGFR, lead to increased goblet cell mucin production and metaplasia [47,48]. The present findings could therefore be of particular importance in asthmatic and COPD subjects, who may experience exacerbations after exposure to particulate matter air pollution [1,14].

## Conclusion

The present investigation suggests that diesel exhaust induced bronchial epithelial inflammatory responses are mediated by the EGFR. The enhanced EGFR expression and phosphorylation of the autophosphorylation site tyr1173 by diesel exhaust is in accordance with the previously demonstrated activation of the JNK, AP-1, p38 MAPK and NFkB pathways and their associated downstream signalling and cytokine production. We could not identify any effect on the MEK and ERK pathways, suggesting that at this 6 hour post exposure time point there was no proliferative/differentiating signalling in the bronchial epithelium. The involvement of EGFR in the airway response to diesel exhaust could potentially be of even more importance in subjects with asthma and COPD in which this receptor tyrosine kinase has been indicated to play a major role in the inflammatory, proliferative and remodelling processes.

## Methods

### Study design

Fifteen non-atopic, non-smoking healthy subjects (11 males, 4 females) mean age; 24 years (range 21–28 years) were included. All had normal lung function, negative skin prick tests against common airborne allergens and were free from respiratory tract infections for at least 6 weeks prior to or during the study period. Each subject was exposed in a chamber to filtered air or DE for one hour, on two separate occasions in a single blind randomized sequence, at least 3 weeks apart, according to a previously described standard protocol [10,49]. During DE exposure, the concentration of particulates with a mass median diameter of less than 10 μm (PM_10_) was kept at 300 μg/m^3^. As a consequence associated pollutants were at the following concentrations: NO_2 _1.6 ppm, NO 4.5 ppm, CO 7.5 ppm, total hydrocarbons 4.3 ppm, formaldehyde 0.26 mg/m^3 ^and suspended particulates 4.3 × 10^6 ^cm^3^. During exposure the subjects alternated rest and moderate exercise (minute ventilation [V_E_] = 20 L/min/m^2^) on a bicycle ergometer at 15-minute intervals. The study was performed according to the Declaration of Helsinki and was approved by the local ethics committee. All subjects gave their written informed consent.

### Bronchoscopy and processing of biopsies

Bronchoscopy with endobronchial biopsy sampling in alternating lungs between the two occasions was performed six hours after the end of the exposure period. Biopsies were fixed in chilled acetone containing protease inhibitors (20 mM iodoacetamide and 2 mM phenyl methyl sulfonyl fluoride) and kept at -20°C overnight (16–20 hours). The day after biopsy sampling, the biopsies were processed into glycolmethacrylate (GMA) resin, as previously described [50]. The GMA embedded biopsies were stored in airtight containers at -20°C until used for cutting and immunostaining with primary antibodies given in table 2. From each subject and exposure, two sections from one biopsy with proper morphologic structure were cut at 2 μm thickness. IgG and tris buffere saline with 0.5% triton-x-100 (TBST) with 1% BSA was used as negative controls (figure 1E). The endogenous peroxidase were inhibited using 0.1% sodium azide and 0.3% hydrogen peroxide in distilled water. After 3 × 5 minute washes in 0.1% TBST, non-specific antibody binding were blocked with undiluted culture medium containing 10% fetal calf serum and bovin serum albumin (BSA) followed by another blocking step with rabbit norma serum or swine normal serum for 30 minute each blocking step. Primary antibody (Table 2) were applied and incubated overnight, biotinylated rabbit anti mouse were used as secondary antibody on slide stained with mAb, and biotinylated swine anti rabbit antibody were applied on slides immunostained with primary rabbit antibodies. Streptavidine-biotin horseradish-peroxidase complex followed by diaminobenzidine were used to visualize the immunoractivity [15].

**Table 2:** Antibodies used for immunohistochemical staining

**Antibody**	**Clone or Source**	**Dilution**	**Specificity against**	**Source**
EGFR	H11Mouse	1:140	EGFR	Dako Glostrup, Denmark
P-Tyr 845	Rabbit	1:45	Phosphorylated Tyr 845on EGFR	Cell Signaling Technology, MA, USA
P-Tyr 992	Rabbit	1:30	Phosphorylated Tyr 992on EGFR	Cell Signaling Technology, MA, USA
P-Tyr 1068	1H12Mouse	1:45	Phosphorylated Tyr 1068on EGFR	Cell Signaling Technology, MA, USA
P-Tyr 1110	Rabbit	1:30	Phosphorylated Tyr 1110on EGFR	Santa Cruz Biotechnology, Santa Cruz, CA, USA
P-Tyr 1173	Rabbit	1:60	Phosphorylated Tyr 1173on EGFR	Santa Cruz Biotechnology, Santa Cruz, CA, USA
P-Tyr 416	Rabbit	1:45	Phosphorylated Tyr 416on Src family	Cell Signaling Technology, MA, USA
P-MEK 1, 2	Rabbit	1:40	Phosphorylated Ser 217/221	Cell Signaling Technology, MA, USA
P-ERK 1, 2	Rabbit	1:40	Phosphorylated Thr 202 and Tyr 204 of human ERK	Cell Signaling Technology, MA, USA

### Quantification of immunostaining

The immunoreactivity was quantified using a colour video camera (Sony DXC-950P 3-CCD three-chip power HAD) containing 380 000 effective picture elements (pixels) (Sony, Tokyo Japan). The camera was connected to a LEICA imaging workstation, with highly specific PC software (Leica Q500IW, Leica Cambridge UK). Only areas with intact epithelium were used for quantification. The immunoreactivity was determined as positive staining (figure 1F), and given as percentage of the total epithelial area selected with the image system, as previously reported [15].

### Statistical analysis

Subjects acted as their own controls, and the comparison of post-air and post-DE stainings were performed with Wilcoxon's paired rank test, using SPSS for Windows version 11 (SPSS, Inc., Chicago, IL, USA). A p-value less than 0.05 was considered significant.

## Competing interests

The authors declare that they have no competing interests.

## Authors' contributions

JP came up with the study concept, wrote the manuscript and had participated in the design, performance and analyses of the study material. AB was participating in the design, performance and analyses in the study as well as writing the paper. FJK participated in the design of the study, data evaluation as well as the writing the paper. DED was involved in the data interpretation as well as writing the paper. SJW took part in the evaluation of the study data, discussions and the writing of the paper. STH participated in the design of the study, interpretations and finalising the manuscript. TS contributed to the research idea, the design, performance and analyses in the study as well as writing the paper. All authors read and approved of the paper to be published.
